# Exosomal circ_0093708 as a potential ferroptosis biomarker in cerebral ischemia–reperfusion injury

**DOI:** 10.3389/fneur.2025.1633393

**Published:** 2025-08-01

**Authors:** Shuyin Ma, Xiaodong Zhang, Jiaxin Fan, Mengying Chen, Qingling Yao, Nan Zhang, Kaili Shi, Shuang Du, Yuxuan Cheng, Huiyang Qu, Minyu Duan, Han Yang, Tiantian Gao, Shuqin Zhan

**Affiliations:** Department of Neurology, The Second Affiliated Hospital of Xi’an Jiaotong University, Xi’an, Shaanxi, China

**Keywords:** exosomes, cerebral ischemia–reperfusion injury, ferroptosis, DUSP1, circular RNA

## Abstract

**Background:**

Ferroptosis plays a critical role in neuronal injury following cerebral infarction. However, effective therapeutic strategies targeting ferroptosis after cerebral ischemia–reperfusion injury (CI/RI) remain limited. Exosome-based therapy holds significant promise in this context. This study aims to identify key exosomal markers of ferroptosis.

**Methods:**

By integrating and analyzing multiple GSE datasets, we identified ferroptosis-associated key genes. These findings were further validated in external databases, cellular models, and animal experiments using malondialdehyde (MDA), glutathione (GSH), iron, reactive oxygen species (ROS) assays, qRT-PCR, Western blotting. By further establishing a ferroptosis model and inhibiting DUSP1 with drugs, we further explored the potential function of DUSP1 in ferroptosis. The role of miR-101-3p was assessed in CI/RI models, while the diagnostic value of exosomal circular RNA was evaluated using receiver operating characteristic curve analysis.

**Results:**

Combined differential analysis revealed PTGS2 and DUSP1 as ferroptosis-associated genes potentially regulated by exosomal circRNAs. In cellular and animal models, ferroptosis post-CI/RI was confirmed by elevated MDA, iron, and ROS levels, alongside reduced GSH. DUSP1 expression was significantly upregulated during ferroptosis, as demonstrated by qRT-PCR, Western blotting, and immunofluorescence. In the simple ferroptosis model, the expression of DUSP1 increases and inhibiting DUSP1 can aggravate ferroptosis. Conversely, miR-101-3p was downregulated in CI/RI, consistent with database predictions. Notably, exosomal circ_0093708 exhibited high diagnostic accuracy (Area under the curve = 0.93, sensitivity = 90%, specificity = 90%). Bioinformatics analysis suggested binding interactions among circ_0093708, miR-101-3p, and DUSP1.

**Conclusion:**

Exosomal circ_0093708 is linked to DUSP1 and PTGS2 expression by sponging miR-101-3p, positioning it as a potential biomarker for ferroptosis in CI/RI.

## 1 Introduction

Stroke, a leading cause of global morbidity and mortality, poses severe threats to patient survival and often results in long-term disability among survivors (1). Furthermore, the incidence of stroke among young people has increased (2). Ischemic stroke (IS), accounting for the majority of stroke cases, is distinguished from other subtypes such as hemorrhagic stroke (3). Current clinical interventions, including intravenous thrombolysis and mechanical thrombectomy, are widely employed to restore cerebral blood flow (4). However, recanalization may paradoxically exacerbate brain damage through secondary injury mechanisms, particularly after prolonged ischemia. The cerebral ischemia–reperfusion injury (CI/RI) is driven by pathological processes such as apoptosis, the inflammatory response, oxidative stress, extracellular matrix remodeling, angiogenesis, cell hypertrophy, fibrosis, neurogenesis, blood–brain barrier damage and ferroptosis (5). Notably, ferroptosis—an iron-dependent form of regulated cell death characterized by excessive accumulation of iron ions and lipid peroxidation (6)—has been implicated as a critical contributor to CI/RI. Thus, targeting ferroptosis may represent a promising therapeutic strategy to mitigate neurodegeneration following CI/RI.

Exosomes, bilayer lipid membrane vesicles secreted by cells, facilitate intercellular communication by transporting nucleic acids, proteins, and lipids across biological barriers, including the blood–brain barrier (7). Among their cargo, circular RNAs (circRNAs) are notably abundant and stable, functioning as competitive endogenous RNAs (ceRNAs) that sequester microRNAs (miRNAs) to regulate gene expression. Recent studies reveal altered circRNAs profiles in plasma exosomes from IS patients (8), with some exhibiting diagnostic potential for large-artery atherosclerotic IS (9). However, whether exosomal circRNAs participate in ferroptosis during CI/RI, or serve as biomarkers remains unclear.

This study aims to identify ferroptosis-associated markers in plasma exosomes through integrated bioinformatics analysis, decipher the downstream miRNA-mRNA regulatory networks involved in CI/RI-induced ferroptosis, and explore novel diagnostic biomarkers for ferroptosis after CI/RI. By exploring the possible targets of plasma exosomal circRNAs in ferroptosis after CI/RI, this study may promote the understanding of the pathogenesis of CI/RI and stimulate targeted intervention measures.

## 2 Materials and methods

### 2.1 Data sources

We obtained discovery datasets GSE16561 (10) and GSE195442 (8) and validation datasets GSE110993 (11) and GSE22255 (12) from the GEO database.

The GSE16561 dataset included 39 cerebral infarction patients and 24 healthy controls, while GSE195442 comprised 10 patients and 10 controls. For validation, GSE110993 contained 20 cerebral infarction patients and 20 healthy controls, and GSE22255 included 20 patients and 20 controls. Detailed characteristics of these datasets are presented in Table 1.

**Table 1:** Detailed information on the studied gene expression datasets.

Dataset	Platform	Samples	IS	Control	Application
GSE16561	GPL6883	Whole blood	39	24	Identification of DEmRNAs
GSE195442	GPL31275	Plasma exosomes	10	10	Identification of DEcircRNAs
GSE110993	GPL15456	Peripheral blood	20	20	Validation for DEmiRNAs
GSE22255	GPL570	Peripheral blood	20	20	Validation for key biomarkers

### 2.2 Data preprocessing and identification of DEmRNAs, DEmiRNAs, and DEcircRNAs

Following normalization of raw data, differential expression analysis was performed using the R Bioconductor limma package (v3.52.3). Genes meeting the criteria of *p* < 0.05 and |log2fold change| > 0.58 were identified as differentially expressed mRNAs (13), while circRNAs with *p* < 0.05 and |log2fold change| > 1.2 were classified as differentially expressed. Subsequently, DEcircRNAs were analyzed using the circbank database^1^ (14) to predict potential miRNA interactions. The resulting miRNAs were further filtered based on differential expression (*p* < 0.05 and |log2 fold change| > 1.2) using GSE110993 data. Heatmaps and volcano plots were generated using the R pheatmap package (v1.0.12).

### 2.3 Construction of the ceRNAs network

Predicted miRNAs from the circbank database (see text footnote 1) were validated against GSE110993 data. miRNA-mRNA interaction datasets were obtained from miRcode (v6.0, http://www.mircode.org/), miRTarBase (v8.0, http://mirtarbase.mbc.nctu.edu.tw/), and TargetScan (v7.2, http://www.targetscan.org/), with potential interactions identified using Perl software (v5.36.0, https://www.perl.org/). Only mRNA-miRNA pairs confirmed by all three databases were included in subsequent analyses.

### 2.4 Screening of key differentially expressed genes of ferroptosis

Using cerebral infarction diagnosis as the dependent variable, we analyzed 21 DEGs identified through intersection analysis of GSE16151 dataset and GSE110993 predictions. Ferroptosis-associated genes (including drivers, suppressors, markers, and unclassified genes) were retrieved from FerrDb (15).^2^ Key ferroptosis DEGs were visualized using the R “Venn” package (v1.11).

### 2.5 Cell culture and oxygen–glucose deprivation/reperfusion model

PC12 cells (rat pheochromocytoma cell line; Shanghai Institute of Biological Science, Chinese Academy of Sciences) were maintained in high-glucose DMEM (C11995500BT, Gibco, China) containing 10% fetal bovine serum (13011-8611, TIANHANG, China), along with 100 U/mL penicillin and 100 μg/mL streptomycin (C100C5, NCM Biotech, China), at 37°C under 5% CO₂ humidified conditions. To establish the OGD/R model, cells were subjected to incubation in glucose/serum-free DMEM (11,966,025, Gibco) using a tri-gas incubator (37°C, 94% N₂/5% CO₂/1% O₂) for 4 h, followed by a 24 h recovery period in complete DMEM. The experimental design comprised two groups: Control (standard culture conditions) and OGD/R.

### 2.6 Cell viability assay

PC12 cells (10,000 cells/well) were seeded in 96-well plates with 100 μL complete medium. After OGD/R, cells were incubated with 10 μL CCK-8 reagent (G1613-5ML, Servicebio, China) in 100 μL fresh DMEM for 1 h at 37°C. Cell viability was calculated as: [(Aexperimental − Ablank)/(Acontrol − Ablank)] × 100%.

### 2.7 Intracellular reactive oxygen species detection

The ROS were measured using dihydroethidium (DHE; CA1420, Solarbio, China). Cells were incubated with DHE (1:1000 dilution in DMEM) at 37°C for 30 min in the dark, washed with PBS (G4202-500, Servicebio, China), and visualized using an inverted fluorescence microscope (DMi8, Leica).

### 2.8 Biochemical assays

The content of intracellular iron content, malondialdehyde (MDA), and reduced glutathione (GSH) levels were quantified using commercial kits (BC5315, BC0025, and BC1175 respectively; Solarbio, China) following manufacturer protocols. Tissue samples from ischemic penumbra were processed identically. Protein content was determined using a BCA kit (BL521A, Biosharp, China), with absorbance measurements normalized to protein concentration.

### 2.9 RNA extraction and quantitative real-time PCR

Total RNA was extracted from both cultured cells and brain tissue samples using RNAiso Plus reagent (9,109, Takara, Japan). Reverse transcription was performed using HiScript II Reverse Transcriptase (R223, Vazyme, China) following the manufacturer’s protocol. qRT-PCR amplification was carried out using ChamQ Universal SYBR qPCR Master Mix (Q311, Vazyme, China). The miRNA was reverse transcribed using the miRNA 1st Strand cDNA Synthesis Kit (by stem-loop) (MR101-01, Vazyme, China), and quantified using the miRNA Universal SYBR qPCR Master Mix (MQ101-01, Vazyme, China). All reactions were performed on a CFX Opus Deepwell Real-Time PCR Detection System (Bio-Rad, United States). GAPDH was used as the endogenous reference gene, and relative gene expression levels were calculated using the 2^−ΔΔCt^ method. The primer sequences used in this study are provided in Table 2.

**Table 2:** The sequence of primers.

Gene	Species	Primer name	Sequence (5′-3′)
DUSP1	Rat	FP	CGCTCCTTCTTCGCCTTCAAC
		RP	CGTTCGTCCAGCAGCACTAC
GAPDH	Rat	FP	AAGTTCAACGGCACAGTCAAGG
		RP	GACATACTCAGCACCAGCATCAC
TFRC	Rat	FP	TGGGTCTAAGTCTACAATGGCTG
		RP	CCCTCATGACGAATCTGTTTG
GPX4	Rat	FP	ACCAGTTCGGGAGGCAGGAG
PTGS2	Rat	RP	CACAGTGGGTGGGCATCGTC
		FP	TTCCTCCTGTGGCTGATGACTG
		RP	AGGTCCTCGCTTCTGATCTGTC
miR-101-3p	Rat	FP	GCGCGCGTACAGTACTGTGATA
		RP	ATCCAGTGCAGGGTCCGAGG
		RT	GTCGTATCCAGTGCAGGGTCCG AGGTATTCGCACTGGATACGACTTCAGT
U6	Rat	FP	CTCGCTTCGGCAGCACA
		RP	AACGCTTCACGAATTTGCGT
		RT	AACGCTTCACGAATTTGCGT

### 2.10 Drug administration

CIL56 is a selective ferroptosis inducer that can induce ferroptosis by generating iron-dependent ROS. We chose CIL56 (T4309, TargetMol, America) to construct the ferroptosis model under the condition of 0.7 μM for 24 h.

(E)-2-benzylidene-3-(cyclohexylamino)-2,3-dihydro-1H-inden-1-one (BCI) is a commonly used inhibitor of MKP-1. We used BCI (T10486, TargetMol, America) to inhibit DUSP1 in PC12 cells under the condition of 10 μM for 24 h.

### 2.11 Animals and middle cerebral artery occlusion/reperfusion model

**Figure 1:**
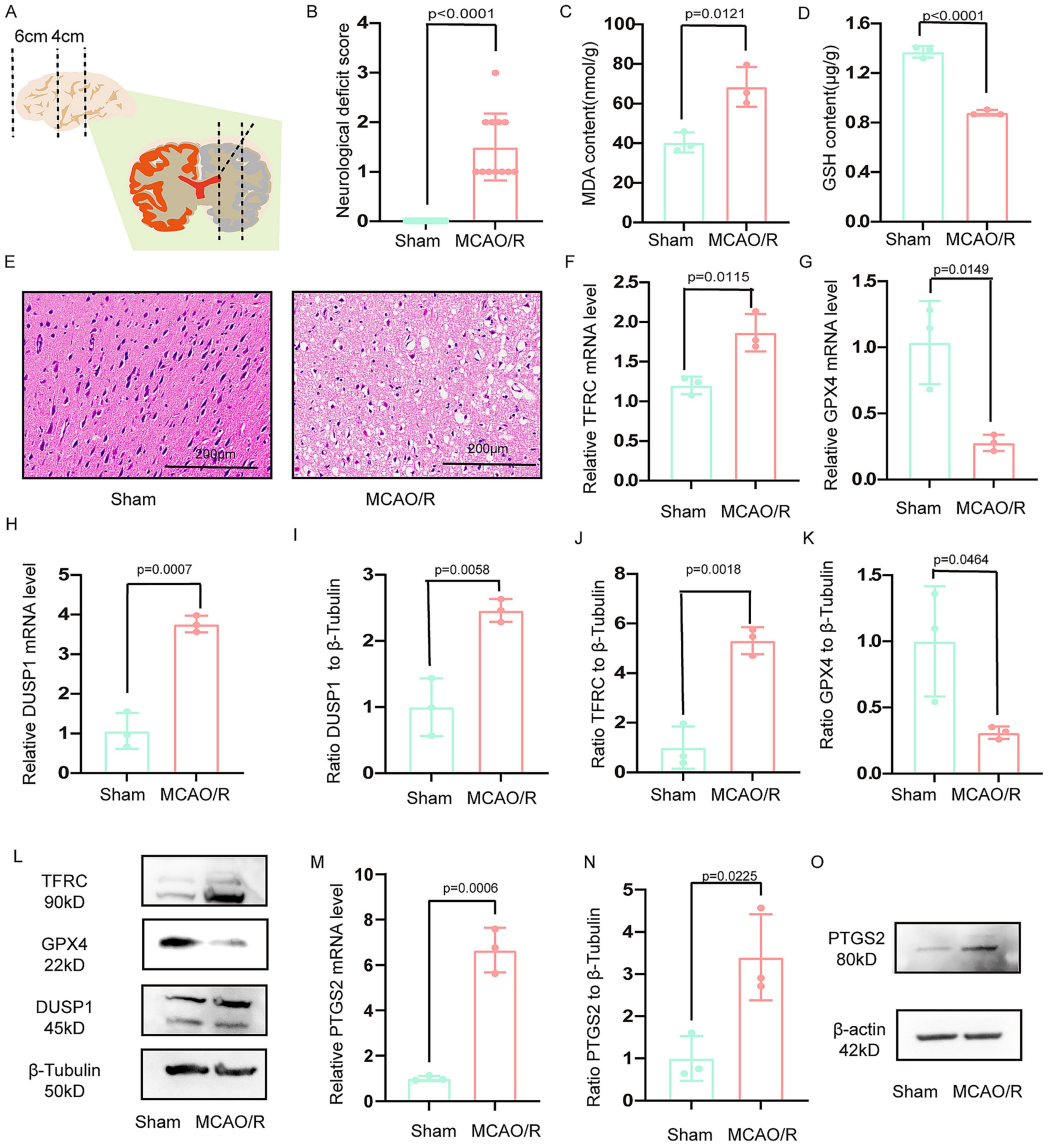
Verification the DUSP1 and PTGS2 in the vitro model. **(A)** Schematic representation of the ischemic penumbra sampling area. **(B)** Neurological deficit scores in MCAO/R rats. **(C,D)** Glutathione (GSH) and malondialdehyde (MDA) levels in the ischemic penumbra. **(E)** Histopathological changes in the ischemic penumbra (scale bar: 200 μm). **(F,G)** The mRNA expression levels of TFRC, and GPX4 in the ischemic penumbra. **(H)** The mRNA expression levels of DUSP1 in the ischemic penumbra. **(I–L)** The Western blot of DUSP1, TFRC and GPX4 in the ischemic penumbra. **(M)** The mRNA expression levels of PTGS2 in the ischemic penumbra. **(N,O)** The Western blot of PTGS2 in the ischemic penumbra. Except for the data in **(B)** which underwent the Mann–Whitney U test, the remaining data underwent the two independent sample *t*-tests after normality tests. Data are presented as mean ± SD. MCAO/R, middle cerebral artery obstruction/reperfusion.

Adult male Sprague–Dawley rats (250–280 g; Experimental Animal Center of Xi’an Jiaotong University, License: XJTUAE2024-076) were housed under standard conditions. The MCAO/R was performed under 2% pentobarbital sodium anesthesia (40 mg/kg, i.p.). After exposing the carotid sheath, the common carotid artery and external carotid artery were ligated. A nylon monofilament (2636A4, Beijing Cinontech, China) was inserted 18–20 mm into the internal carotid artery to occlude the middle cerebral artery for 90 min, followed by reperfusion. Sham-operated rats underwent identical procedures without filament insertion. Twelve rats were included in each group (sham and MCAO/R), with animals dying before the 24 h endpoint being excluded and replaced sequentially. Ischemic penumbra sampling followed established protocols (16), as illustrated in Figure 1A.

### 2.12 Neurological deficit assessment

Neurological function was assessed 24 h after surgery using the modified Zea Longa 5-point scale (0 = no deficit; 4 = involuntary movement with consciousness disturbance). Animals were excluded if they: (1) scored 0 (model failure) or 4 (excessive injury), (2) showed subarachnoid hemorrhage at necropsy, or (3) died before the sampling endpoint.

### 2.13 Histopathological analysis

At 24 h post-reperfusion, rats were perfused transcardially with PBS followed by 4% paraformaldehyde. Brains were dehydrated, paraffin-embedded, and sectioned (4 μm) for hematoxylin and eosin staining and histopathological examination.

### 2.14 Western blotting

Protein extraction was conducted using RIPA lysis buffer (PL001, ZHHC, China) supplemented with protease inhibitors. Protein concentrations were quantified using a BCA assay (BL521A, Biosharp, China), with 30 μg of protein per sample mixed with 5 × loading buffer (ZS306-1, Zomanbio, China) at a 4:1 ratio. Following denaturation (100°C, 5 min), samples were resolved on 10% SDS-PAGE gels (PG212, Epizeme, China) and transferred to PVDF membranes (ISEQ00010, Millipore, United States) at 300 mA for 90 min. Membranes were blocked with RapidBlock solution (PS108, Epizeme, China) for 15 min at room temperature, followed by overnight incubation at 4°C with primary antibodies diluted in antibody dilution buffer (PS119, Epizeme, China). The following primary antibodies were used: DUSP1 (ET1701-82, Huabio, China; 1:1000), GPX4 (ET1706-45, Huabio, China; 1:10000), TFRC (ab269513, Abcam, United Kingdom; 1:5000), *β*-tubulin (66240-1-lg, Proteintech, China; 1:20000), PTGS2 (TP53818, Abmart, China,1:500), β-actin (66009-1-Ig, Proteintech, China; 1:20000). After TBST washes, membranes were incubated with HRP-conjugated secondary antibodies (SA00001-1/SA00001-2, Proteintech, China; 1:10000) for 1 h at room temperature. Protein bands were visualized using a chemiluminescence imaging system (GelView 6000Plus, BLTLUX, China). For reprobing, membranes were stripped with antibody stripping solution (WB6500, NCM Biotech, China). Band intensities were quantified using ImageJ software (v1.53t, National Institutes of Health, United States).

### 2.15 Statistical analysis

**Figure 2:**
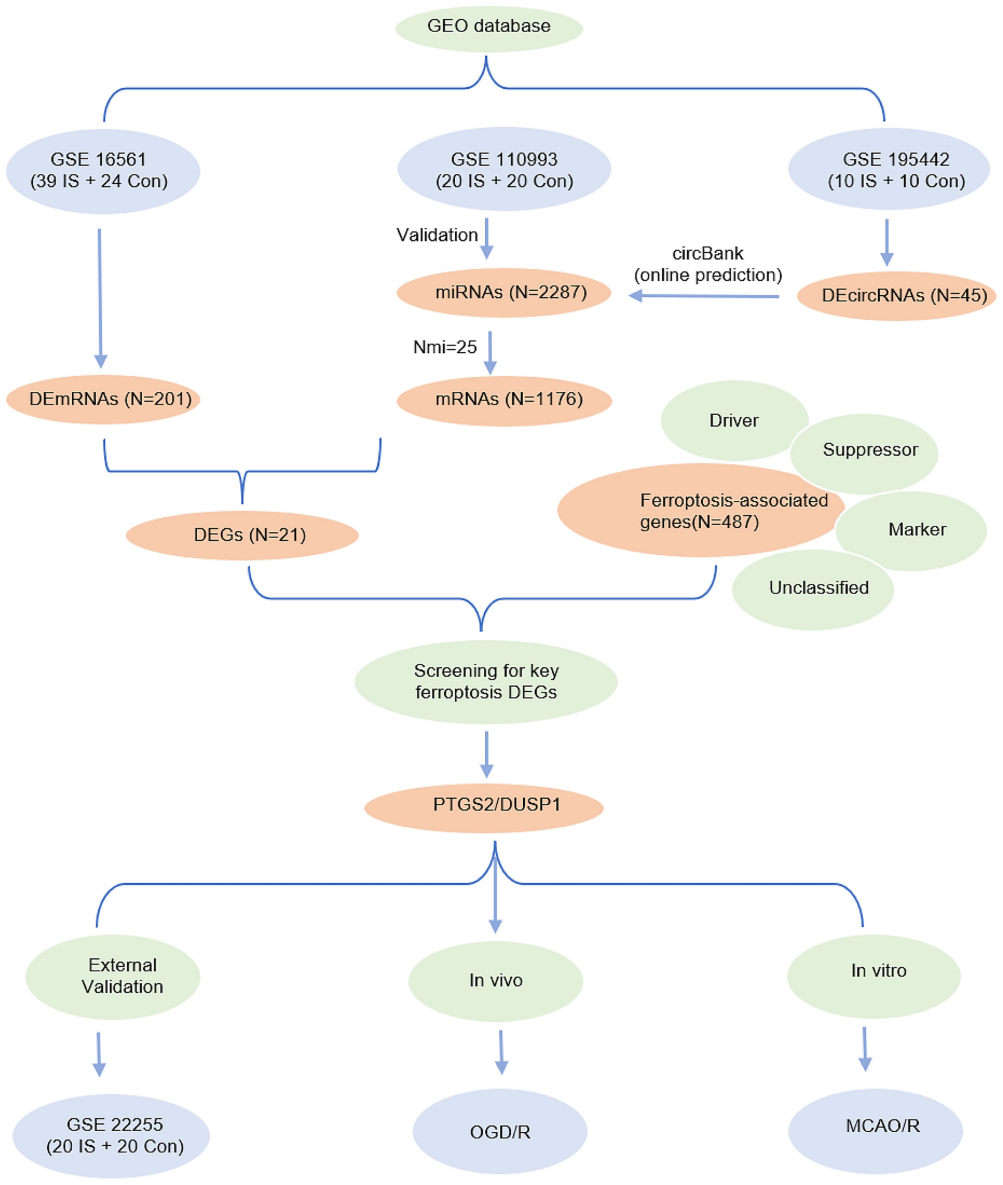
Entire working processes of the study. IS, ischemic stroke; Con, controls; DEGs, differently expressed genes; OGD/R, oxygen–glucose deprivation/reperfusion; MCAO/R, middle cerebral artery obstruction/reperfusion.

Statistical analyses were performed using R software (v4.3.0, https://www.r-project.org/) and GraphPad Prism (v9.0, San Diego, California, United States). Continuous variables with normal distribution were compared using Student’s *t*-test, while non-normally distributed data were analyzed using the Mann–Whitney U test. Brown-Forsythe tests with Dunnett’s T3 *post hoc* correction were used for multiple comparisons. A *p*-value < 0.05 was considered statistically significant. The study flowchart is presented in Figure 2.

## 3 Results

### 3.1 Identification of differentially expressed mRNAs and circRNAs

**Figure 3:**
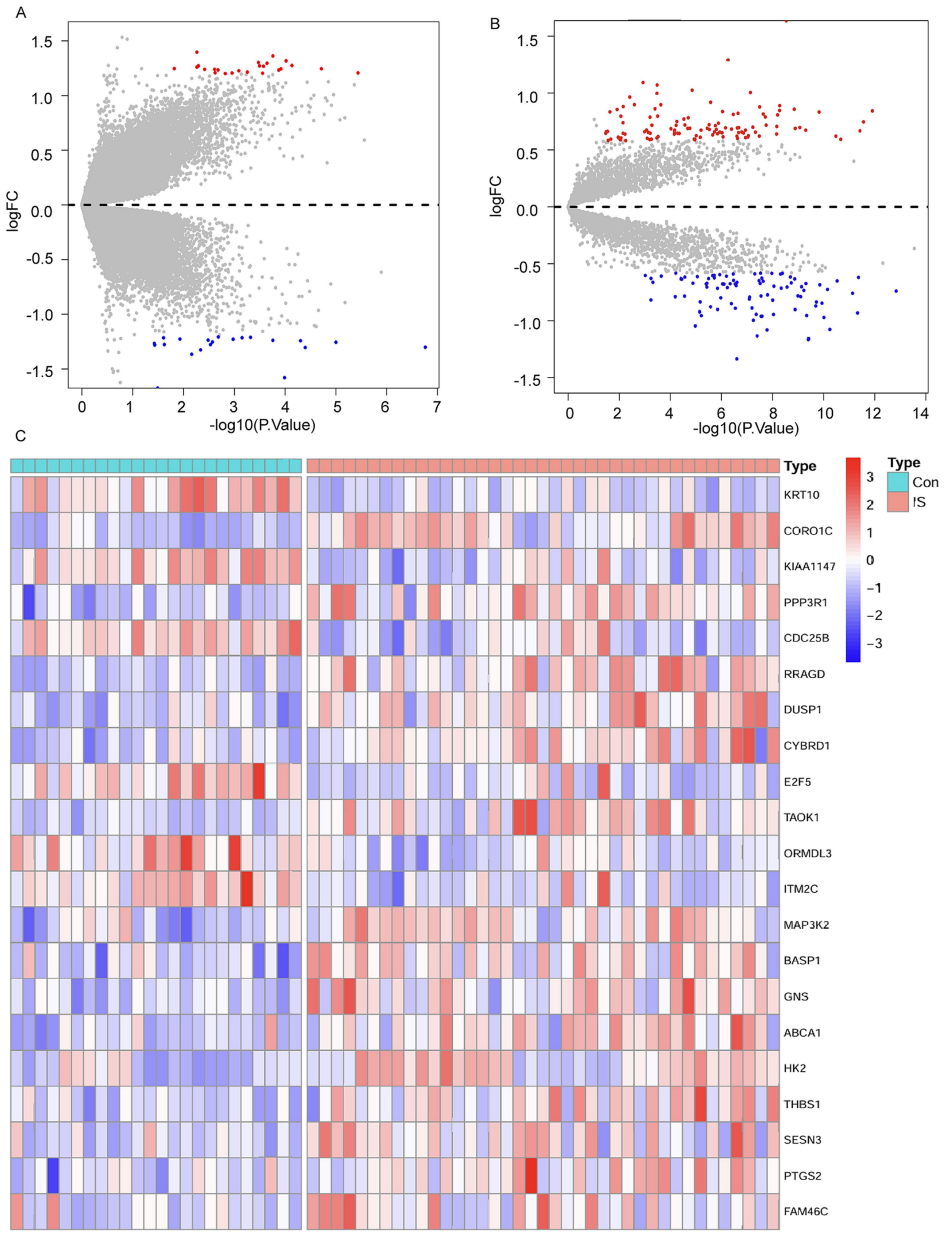
Differential expression analysis. **(A)** Volcano plot for DEcircRNAs in the GSE195442 dataset. **(B)** Volcano plot for DEmRNAs in the GSE16561 dataset. **(C)** Cluster heatmap for 21 DEmRNAs in the GSE16561 dataset.

Through comprehensive analysis of the GSE195442 and GSE16561 datasets, we identified 45 differentially expressed circRNAs (DEcircRNAs) and 201 differentially expressed mRNAs (DEmRNAs), respectively. The expression patterns of these differentially regulated circRNAs and mRNAs were clearly visualized through volcano plots (Figures 3A,B). Further intersection analysis between the DEmRNAs from GSE16561 and the potential mRNA targets predicted by miRNAs in GSE110993 revealed 21 overlapping genes. The expression profiles of these 21 DEmRNAs across different samples were subsequently illustrated using a heatmap (Figure 3C).

### 3.2 Mining of key ferroptosis genes and external validation

**Figure 4:**
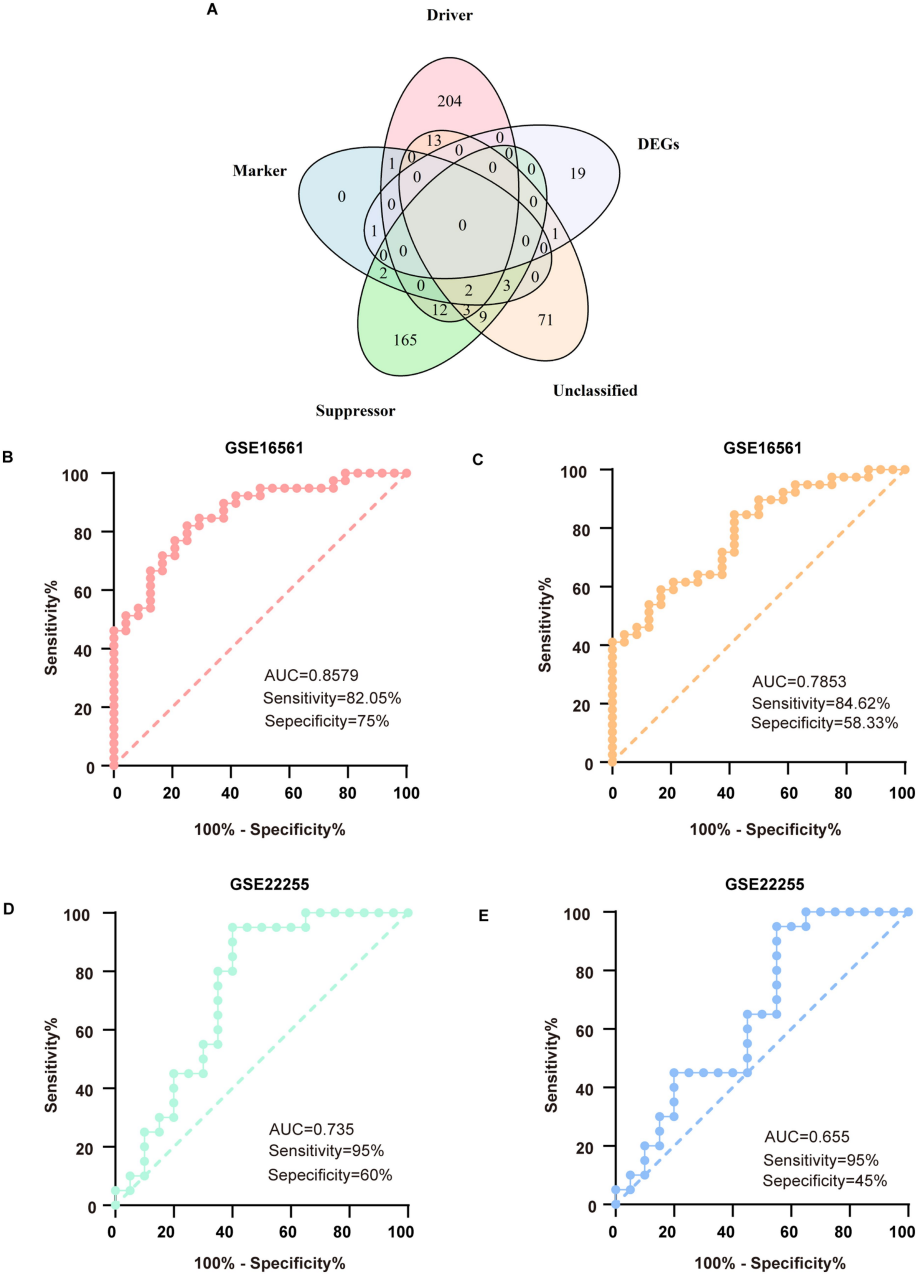
Mining of key genes for ferroptosis and verification in external databases. **(A)** The 5-set Venn diagram showing the comprehensive strategy among suppressor ferroptosis-associated genes (green circle), unclassified ferroptosis-associated genes (orange circle), marker ferroptosis-associated genes (blue circle), driver ferroptosis-associated genes (pink circle), and differentially expressed genes (DEGs) (purple circle). **(B)** ROC analysis for DUSP1 in the discovery dataset. **(C)** ROC analysis for PTGS2 in the discovery dataset. **(D)** ROC analysis for DUSP1 in the validation dataset. **(E)** ROC analysis for PTGS2 in the validation dataset. ROC, receiver operating characteristic.

From the FerrDb database, we compiled a comprehensive set of 487 ferroptosis- associated genes, consisting of 235 driver genes, 196 suppressor genes, 102 unclassified genes, and 9 marker genes. By intersecting these genes with our 21 DEmRNAs, we identified two candidate ferroptosis-associated genes: the ferroptosis marker gene PTGS2 and the unclassified gene DUSP1 (Figure 4A). In the GSE16561 dataset, DUSP1 demonstrated robust diagnostic potential, with an area under the curve (AUC) of 0.8579, sensitivity of 82.05%, and specificity of 75% (Figure 4B). PTGS2 showed moderate diagnostic performance, with an AUC of 0.7853, sensitivity of 84.62%, and specificity of 58.33% (Figure 4C). These findings were further validated in the external dataset GSE22255, where DUSP1 maintained significant diagnostic value (AUC = 0.735, sensitivity = 95%, specificity = 60%; Figure 4D), while PTGS2 exhibited slightly reduced but still notable efficacy (AUC = 0.655, sensitivity = 95%, specificity = 45%; Figure 4E).

### 3.3 *In vitro* validation of DUSP1 and PTGS2 in the OGD/R model

**Figure 5:**
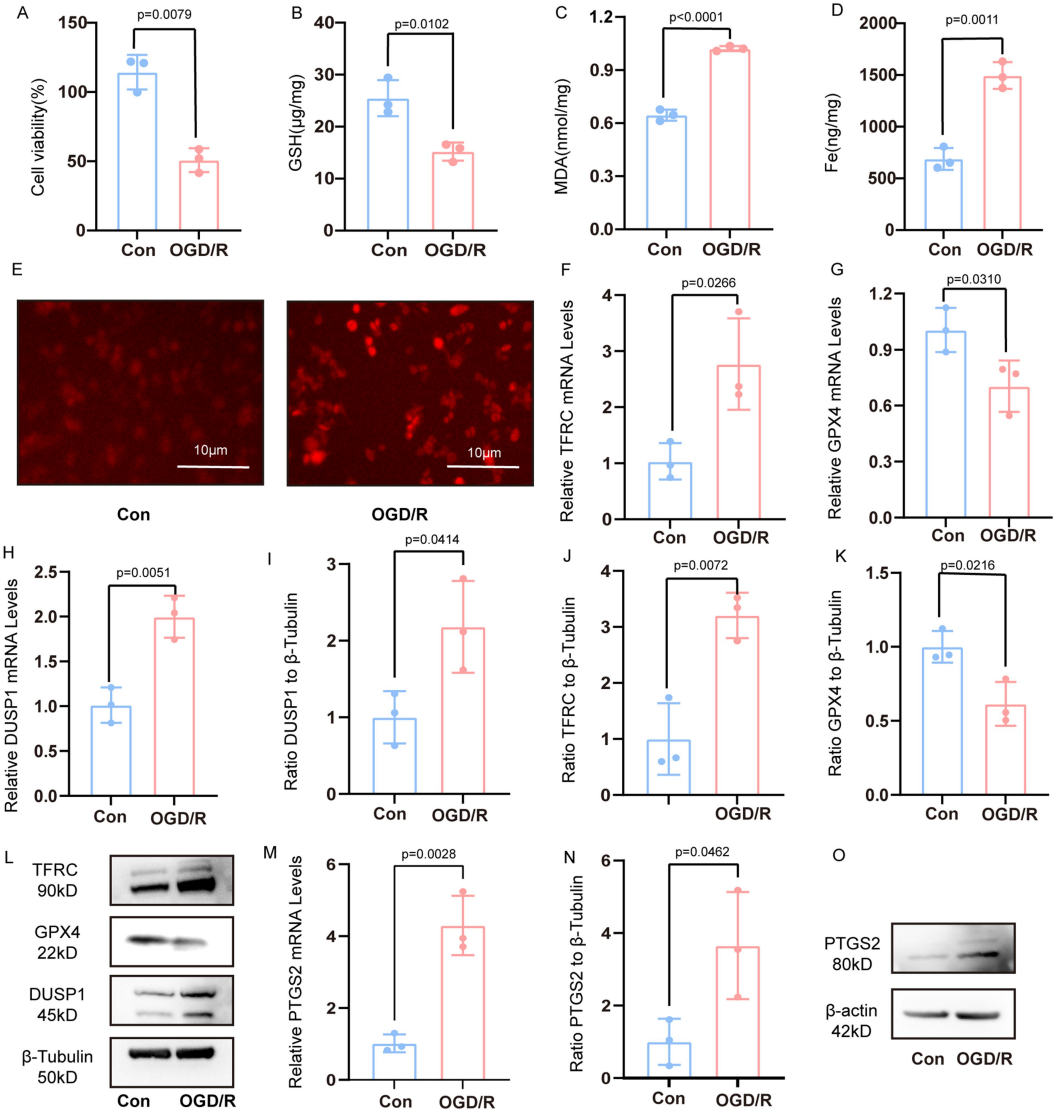
Verification the DUSP1 and PTGS2 in the vivo model. **(A)** Cell viability of PC12 cells after OGD/R treatment. **(B–D)** The content of GSH, MDA and Fe. **(E)** The content of ROS in PC12 cells after OGD/R (scale bars: 10 μm). **(F,G)** The mRNA expression levels of TFRC, and GPX4 in PC12 cells after OGD/R. **(H)** The mRNA expression levels of DUSP1 in PC12 cells after OGD/R. **(I–L)** The Western blot of DUSP1, TFRC, GPX4 in PC12 cells after OGD/R. **(M)** The mRNA expression levels of PTGS2 in PC12 cells after OGD/R. **(N,O)** The Western blot of PTGS2 in PC12 cells after OGD/R. All data underwent two independent sample *t*-tests after normality tests. Data are presented as mean ± SD. Con, controls; OGD/R, oxygen–glucose deprivation/reperfusion.

Following OGD/R treatment in PC12 cells, we observed a marked reduction in cell viability (Figure 5A). Concurrently, characteristic indicators of ferroptosis were evident, including decreased intracellular reduced GSH levels, elevated MDA and iron content, and increased ROS production (Figures 5B–E). The result of qRT-PCR further revealed upregulated TFRC and downregulated GPX4 expression (Figures 5F,G). The mRNA expression of DUSP1 was increased (Figure 5H). The result of Western blotting corroborated these findings at the protein level, demonstrating increased TFRC and decreased GPX4 levels, and significantly elevated DUSP1 protein expression (Figures 5I–L).

Meanwhile, the mRNA expression of PTGS2 was increased (Figure 5M). The result of Western blotting demonstrated that the protein expression of PTGS2 was increased (Figures 5N,O).

### 3.4 *In vivo* validation of DUSP1 and PTGS2 in the MCAO/R model

Successful establishment of the MCAO/R model in rats was confirmed through both behavioral and molecular assessments (Figure 1B). Consistent with the *in vitro* findings, the MCAO/R group showed decreased GSH and increased MDA levels in the ischemic penumbra (Figures 1C,D). The ischemic penumbra region, delineated in Figure 1A, exhibited significant pathological alterations, including neuronal shrinkage, reduced cell density, and vacuolar degeneration (Figure 1E). Molecular analyses revealed upregulated transcription of DUSP1 and TFRC, coupled with downregulated GPX4 expression (Figures 1F–H). Western blotting confirmed these trends at the protein level, with increased TFRC and decreased GPX4, and significantly elevated DUSP1 protein expression (Figures 1I–L).

Meanwhile, the mRNA expression of PTGS2 in ischemic penumbra of rat was increased (Figure 1M). The result of Western blotting demonstrated that the protein expression of PTGS2 in ischemic penumbra was increased (Figures 1N,O).

### 3.5 Inhibition of DUSP1 exacerbates ferroptosis in PC12 cells

**Figure 6:**
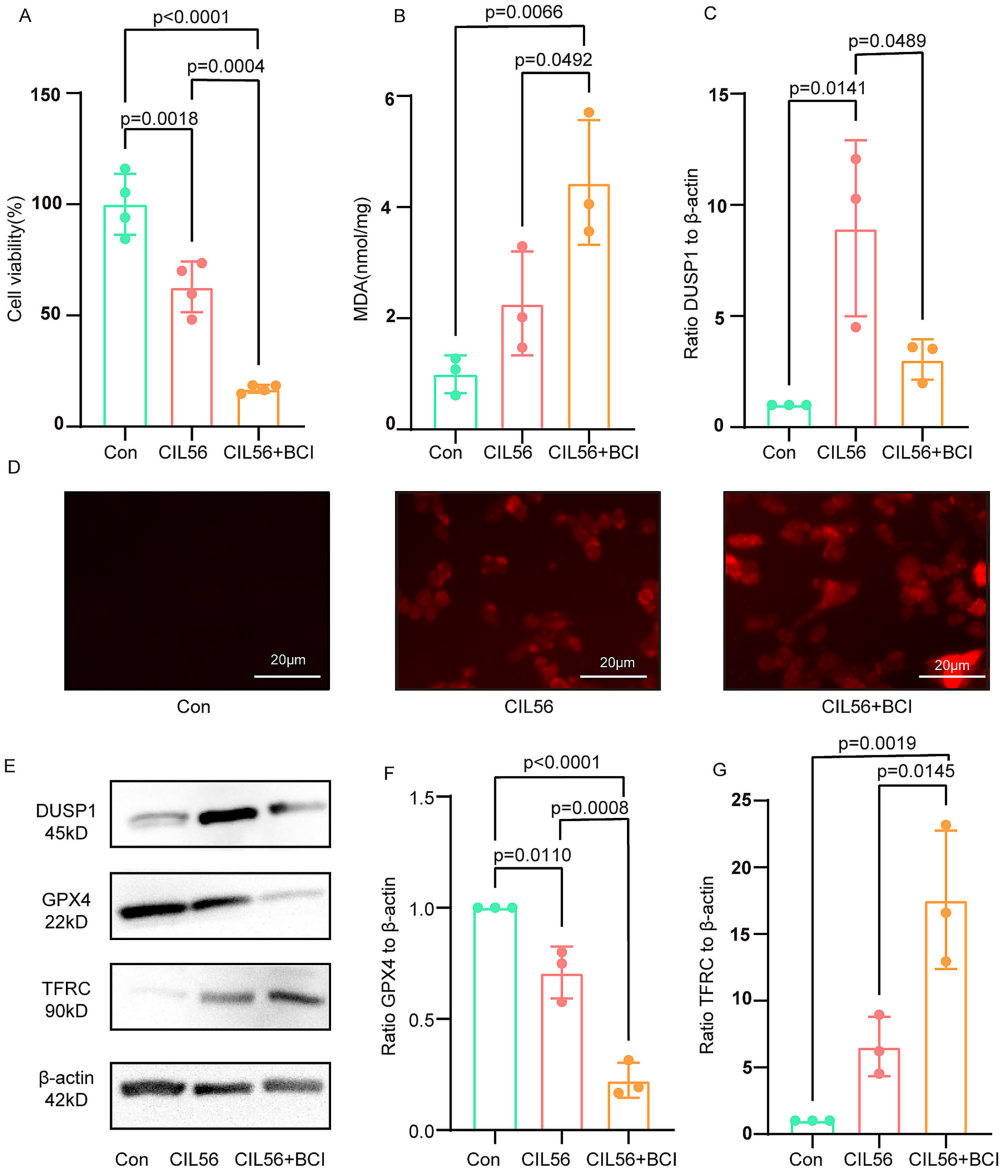
Inhibition of DUSP1 exacerbates ferroptosis in PC12 cells. **(A)** Cell viability after CIL56 treatment. **(B)** Intracellular MDA levels. **(C)** Effect of BCI on DUSP1 inhibition in the ferroptosis model. **(D)** Intracellular ROS levels (scale bar: 20 μm). **(E)** Protein expression of DUSP1, GPX4, and TFRC. **(F)** Quantification of GPX4 levels. **(G)** Quantification of TFRC levels. Data were analyzed by one-way ANOVA with Tukey’s *post hoc* test and presented as mean ± SD. Con, control.

Treatment of PC12 cells with CIL56 resulted in decreased cell viability. Notably, co-treatment with CIL56 and the DUSP1 inhibitor BCI led to a more pronounced reduction in cell survival compared to CIL56 treatment alone (Figure 6A). Following the induction of ferroptosis by CIL56, intracellular MDA levels were significantly elevated. Importantly, the BCI-treated group exhibited even higher MDA content than cells treated with CIL56 alone (Figure 6B). Consistent with these findings, CIL56 treatment increased intracellular ROS levels in PC12 cells. This effect was markedly enhanced when CIL56 was combined with BCI (Figure 6D).

Western blot analysis revealed that CIL56 successfully induced ferroptosis in PC12 cells, as evidenced by decreased GPX4 expression and increased TFRC levels. Interestingly, DUSP1 expression was upregulated in response to CIL56 treatment (Figure 6C). The ferroptosis effects were further aggravated upon BCI co-treatment, with more pronounced changes in both GPX4 and TFRC protein levels compared to the CIL56-only group (Figures 6E–G). These results collectively suggest that pharmacological inhibition of DUSP1 potentiates ferroptosis in PC12 cells, indicating that DUSP1 may function as a negative regulator of ferroptosis.

### 3.6 Verification of miRNA and construction of the ceRNAs network

**Figure 7:**
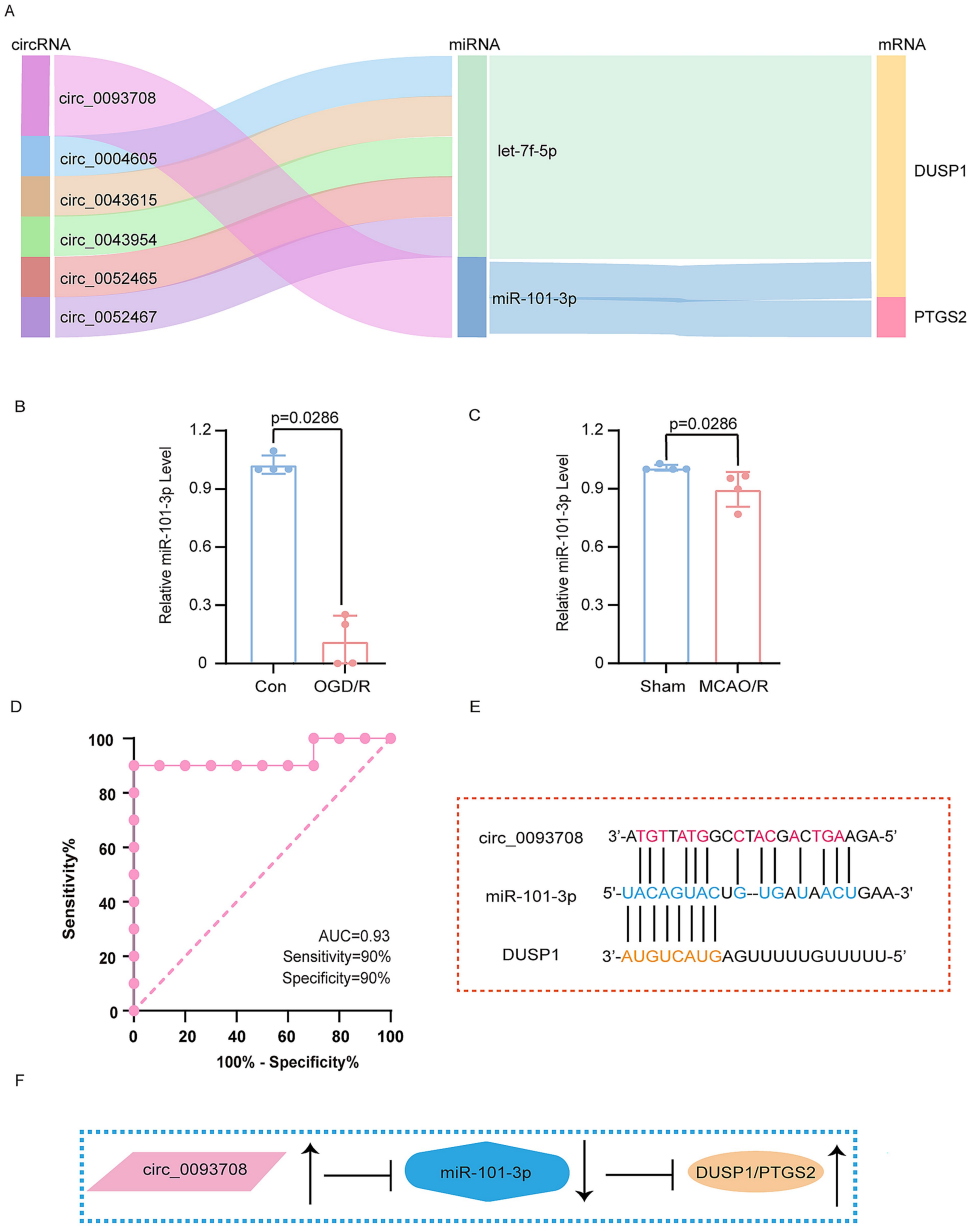
Verification of miRNA and construction of ceRNAs network. **(A)** The Sankey plots of ceRNAs networks. **(B)** The expression of miR-101-3p in OGD/R model. **(C)** The expression of miR-101-3p in MCAO/R model. **(D)** The ROC analysis of circ_0093708 in exosomes. **(E)** The schematic diagram of the binding of circ_0093708, miR-101-3p and DUSP1. **(F)** The schematic diagram of the regulation of circ_0093708, miR-101-3p, DUSP1 and PTGS2. The data in **(B,C)** underwent the Mann–Whitney U test. Con, controls; OGD/R, oxygen–glucose deprivation/reperfusion; MCAO/R, middle cerebral artery obstruction/reperfusion. ROC, receiver operating characteristic.

Through systematic analysis, we constructed a ceRNAs network. Validation using the GSE110993 and GSE195442 datasets confirmed the involvement of these regulatory molecules, as illustrated by the Sankey plots (Figure 7A). Notably, miR-101-3p emerged as a key regulator capable of simultaneously targeting both DUSP1 and PTGS2. Subsequent experiments in both the OGD/R and MCAO/R model demonstrated significant downregulation of miR-101-3p (Figures 7B,C), establishing its role in ferroptosis regulation following CI/RI.

Further investigation revealed that circ_0093708, through competitive binding with miR-101-3p, promotes the expression of both DUSP1 and PTGS2. Remarkably, circ_0093708 exhibited exceptional diagnostic potential for cerebral infarction in the GSE195442 dataset, with an AUC of 0.93, sensitivity of 90%, and specificity of 90% (Figure 7D). The proposed regulatory mechanism of the circ_0093708/miR-101-3p/DUSP1/PTGS2 axis is comprehensively illustrated in Figures 7E,F. This integrated approach not only confirms the central role of DUSP1 in ferroptosis following CI/RI but also identifies a novel ceRNAs network with significant diagnostic and therapeutic potential.

## 4 Discussion

Our study employed bioinformatics approaches to integrate data from multiple databases, constructing a ceRNAs network involving exosomal circRNAs, miRNA, and mRNA. We validated the newly identified ferroptosis-associated gene DUSP1 in external databases, as well as in cellular and animal models. Furthermore, we elucidated the alterations of key miRNAs in these models and demonstrated that exosomal circRNAs could serve as novel biomarkers and potential therapeutic targets for ferroptosis following CI/RI.

Our findings revealed that PTGS2 (prostaglandin-endoperoxide synthase 2, also known as COX2) acts as a downstream target of miR-101-3p and modulates ferroptosis after CI/RI. PTGS2 is a well-established inflammatory mediator that catalyzes prostaglandin synthesis and participates in diverse pathophysiological processes. It is also a confirmed biomarker of ferroptosis, with its mRNA levels positively correlating with ferroptosis sensitivity in various tumor models (17). Post CI/RI, ferroptosis occurs alongside elevated PTGS2 expression (18). Ferroptosis inhibition has been shown to suppress the COX-2/PGE2 pathway (19), while exosomal miR-137 can inhibit COX2/PGE2 activation, conferring neuroprotection (20). Previous study corroborated that PTGS2 levels are increased in the infarcted cortex of MCAO rats. PTGS2 inhibition reduces infarct volume, improves neurological deficit scores, and enhances outcomes (21). Similarly, blocking the p53/PTGS2 pathway mitigates hippocampal neuronal ferroptosis and alleviates CI/RI (22). Collectively, these findings suggest that PTGS2 promotes ferroptosis after CI/RI.

DUSP1, a member of the dual-specificity phosphatase family, dephosphorylates tyrosine and threonine residues, thereby inhibiting mitogen-activated protein kinases (MAPK). In the central nervous system, the MAPK pathway mediates apoptosis, inflammation, autophagy, oxidative stress, and ferroptosis (23–25). The DUSP1 has emerged as an endogenous neuroprotective factor in stroke. Its inhibition exacerbates infarct volume, neurological deficits, and hemorrhagic transformation (26), while its overexpression suppresses the c-Jun N-terminal Kinase (JNK) pathway and neuronal death in OGD/R and MCAO/R models (27). In endothelial cells, DUSP1 overexpression preserves blood–brain barrier integrity by inactivating MAPK, thereby reducing brain injury and improving post-stroke outcomes (28). In ferroptosis research, DUSP1 expression increases upon ferroptosis induction in cancer cell lines (17). Intriguingly, DUSP1 has been identified as a ferroptosis inhibitor in pancreatic cancer, where it blocks lipid peroxidation-dependent ferroptosis (29). Our prior work identified DUSP1 as a reliable oxidative stress marker in ischemic stroke (30). Given its role in reducing ROS-induced cell death and preserving mitochondrial membrane potential (31), DUSP1 likely functions as a ferroptosis suppressor in CI/RI by mitigating ROS accumulation, a hallmark of ferroptosis. Our research results also preliminarily indicate that DUSP1 shows a protective effect in ferroptosis after CI/RI.

Our study demonstrated that miR-101-3p is downregulated in OGD/R and MCAO/R models, as well as in the GSE110993 dataset. This miRNA is a critical regulator in neurological diseases, influencing cerebrovascular and neurodegenerative pathologies (32–35). In human umbilical vein endothelial cells, miR-101-3p mimics induce ROS production and NF-κB activation, whereas its inhibition attenuates endothelial injury, highlighting its role in atherosclerosis (36). In young MCAO mice, miR-101-3p is downregulated but upregulates HDAC9; its overexpression improves neuronal morphology and apoptosis, suggesting a neuroprotective role (37). Conversely, miR-101-3p inhibition enhances DUSP1 expression, suppressing MAPK p38 and NF-κB pathways to reduce inflammation and apoptosis (38). After acute brachial plexus injury, JHDM1D-AS1 targets miR-101-3p to upregulate DUSP1, further underscoring its neuroprotective potential (39). Regarding exactly how miR-101-3p plays a role in CI/RI, more studies are needed for further exploration.

Our research implicates circ_0093708 in exosomes as a potential biomarker for ferroptosis after CI/RI. Growing evidence suggests that exosomal circRNAs can modulate ischemic stroke via ceRNAs network. Exosomal circBBS2 inhibits ferroptosis by targeting miR-494 to activate SLC7A11 signaling in ischemic stroke (40). The circOGDH is elevated in plasma exosomes of acute stroke patients and may mark the ischemic penumbra (41). Hypoxia-preconditioned adipose-derived stem cell exosomes deliver circRps5, promoting M2 microglial polarization and mitigating brain injury (42). circFUNDC1 knockdown alleviates OGD-induced endothelial injury by sponging miR-375 to inhibit PTEN (43). The circ_0000647 interference protects SK-N-SH cells by regulating the miR-126-5p/TRAF3 axis (44). Astrocyte-derived exosomal circSHOC2 reduces neuronal autophagy and apoptosis via the miR-7670-3p/SIRT1 pathway (45). The circRNAs has been increasingly investigated for its critical role in apoptosis, autophagy, angiogenesis, inflammation, oxidative stress, and blood–brain barrier after ischemic stroke by regulating target mRNAs and others, which have a promising future as diagnostic and prognostic biomarkers for ischemic stroke (46).

Our work also has several limitations. First, the binding interactions within the ceRNAs network requires further verification. We will conduct essential experimental validation (e.g., dual-luciferase reporter assay, RNA pull-down followed by qPCR for miR-101-3p, or biotinylated miRNA capture assay) to confirm the direct physical interaction between circ_0093708 and miR-101-3p. Second, circ_0093708 was analyzed bioinformatically but lacks experimental validation of its regulatory role. We plan to verified the effect of circ_0093708 knockdown or overexpression on miR-101-3p levels, DUSP1/PTGS2 expression, ferroptosis markers, and ultimately cell viability in the OGD/R model. Finally, more large-sample clinical data are needed to provide evidence for the clinical effect of circ_0093708. We will develop it in the subsequent work, fully explore the diagnostic value of circ_0093708 in ischemic stroke, and further combine it with the clinical outcomes of patients, in order to provide ideas for the treatment of ischemic stroke.

In summary, our study delineates the involvement of DUSP1 in ferroptosis after CI/RI and explores the circ_0093708/miR-101-3p/DUSP1/PTGS2 ceRNAs axis. Exosomal circ_0093708 holds promise as a diagnostic marker for ferroptosis after CI/RI.

## Data Availability

The datasets presented in this study can be found in online repositories. The names of the repository/repositories and accession number(s) can be found at: https://www.ncbi.nlm.nih.gov/, GSE16561; https://www.ncbi.nlm.nih.gov/, GSE195442; https://www.ncbi.nlm.nih.gov/, GSE110993; https://www.ncbi.nlm.nih.gov/, GSE22255.

## Footnotes

^1^ http://www.circbank.cn/

^2^ http://www.zhounan.org/ferrdb/current
